# Outcomes of Non‐Paroxysmal AF Undergoing Ablation Guided by a Novel High‐Density Panoramic Cycle‐Length Mapping System

**DOI:** 10.1111/pace.70063

**Published:** 2025-10-14

**Authors:** Ting‐Yung Chang, Chin‐Yu Lin, Yenn‐Jiang Lin, Shih‐Lin Chang, Li‐Wei Lo, Yu‐Feng Hu, Fa‐Po Chung, Ling Kuo, Chih‐Min Liu, Cheng‐I. Wu, Shih‐Ann Chen

**Affiliations:** ^1^ Department of Medicine Heart Rhythm Center Division of Cardiology Taipei Veterans General Hospital Taipei Taiwan; ^2^ School of Medicine National Yang Ming Chiao Tung University Taipei Taiwan; ^3^ Institute of Clinical Medicine and Cardiovascular Research Institute National Yang Ming Chiou Tung University Taipei Taiwan; ^4^ National Taipei University of Nursing and Health Sciences Taipei Taiwan; ^5^ Department of Internal Medicine and Cardiovascular Center Taichung Veterans General Hospital Taichung Taiwan; ^6^ Department of Experimental Examination Healthcare and Services Center Taipei Veterans General Hospital Taipei Taiwan; ^7^ Cardiovascular Center Taichung Veterans General Hospital Taichung Taiwan; ^8^ National Chung Hsing University Taichung Taiwan; ^9^ China Medical University Hospital Taichung Taiwan

**Keywords:** ablation, cycle length, non‐paroxysmal atrial fibrillation, outcome, substrate modification

## Abstract

**Background:**

Catheter ablation is a current therapeutic approach for atrial fibrillation (AF). However, the efficacy for non‐paroxysmal AF remains suboptimal.

**Objective:**

We hypothesize that the novel panoramic cycle‐length mapping (CLM) system can guide the pulmonary vein (PV) isolation and additional potential AF drivers.

**Methods:**

A total of 31 patients with non‐paroxysmal AF referred for ablation guided by a new high‐density panoramic CLM system were prospectively enrolled. We then retrospectively screened the patients with non‐paroxysmal AF undergoing the conventional method during the contemporary period (conventional group). They were matched with a 1:2 ratio (31 patients in Group 1 receiving the CLM‐guided approach, and 62 patients in Group 2 receiving the conventional method).

**Results:**

During a mean follow‐up of 23.2 months, patients in Group 1 had fewer recurrent atrial arrhythmias (*p* = 0.037), mainly driven by a reduction in recurrent AF. There was no difference in recurrent atrial tachycardia or atrial flutter. In multivariate analysis, the application of the CLM module was the only independent factor for recurrent atrial arrhythmias after RFCA of non‐paroxysmal AF.

**Conclusions:**

Identification of the potential drivers in non‐paroxysmal AF is crucial. Compared to the conventional method, ablation guided by a new high‐density CLM system might result in better outcomes for patients with non‐paroxysmal AF.

## 1 Introduction

Atrial fibrillation (AF) is the most prevalent sustained cardiac arrhythmia and is associated with substantial morbidity and mortality [1]. AF is perpetuated by stable and rapid reentrant circuits, leading to fibrillatory conduction across the atrial myocardium [2, 3, 4]. Catheter ablation strategies have historically targeted areas exhibiting fractionated electrograms or high‐frequency activity during AF as potential therapeutic sites [4, 5, 6, 7]. However, the clinical efficacy of adjunctive ablation approaches—such as complex fractionated atrial electrogram (CFAE) mapping or linear ablation—following successful circumferential pulmonary vein (PV) isolation remains contentious. Data from the STAR AF II (Substrate and Trigger Ablation for Reduction of Atrial Fibrillation Trial Part II) trial have cast doubt on the incremental benefits of these additional substrate modification techniques [8]. Conversely, the targeted elimination of arrhythmogenic drivers and non‐pulmonary vein (non‐PV) triggers has emerged as a promising strategy, particularly in patients with non‐paroxysmal AF [9]. Notably, recent studies demonstrated that a driver‐targeted substrate modification approach in this patient population yielded superiority over a PV isolation‐only procedure [10]. Nevertheless, the most effective and durable ablation strategy for managing non‐paroxysmal AF remains to be definitively established.

Non‐pulmonary vein (non‐PV) drivers that contribute to the maintenance of non‐paroxysmal AF are characterized by rapid AF cycle lengths (AFCLs), high‐frequency sites, and localized regions exhibiting rotational activation with discernible conduction gradients across the atrial substrate. Previous studies have shown that regions with short cycle lengths (CLs) or higher dominant frequencies (DF) are correlated with PV triggers in paroxysmal AF and AF drivers within the atrial substrate in persistent AF [6, 11]. Emerging evidence indicates that regions where catheter ablation successfully achieves acute termination of persistent AF are often marked by rapid, organized, and regular electrical activity. Furthermore, panoramic mapping studies of AF drivers have identified localized CLs approximating 140 ms, underscoring their potential mechanistic significance [12]. Recently, an innovative cycle‐length mapping (CLM) system has been developed, enabling the quantification of both AFCL and the temporal stability of local electrograms during ongoing AF [13]. The present study aims to evaluate the long‐term efficacy and safety of catheter ablation for non‐paroxysmal AF guided by this advanced CLM system in addition to PV isolation compared with the conventional anatomic approach of PV isolation along.

## 2 Method

### 2.1 Study Population

**FIGURE 1:**
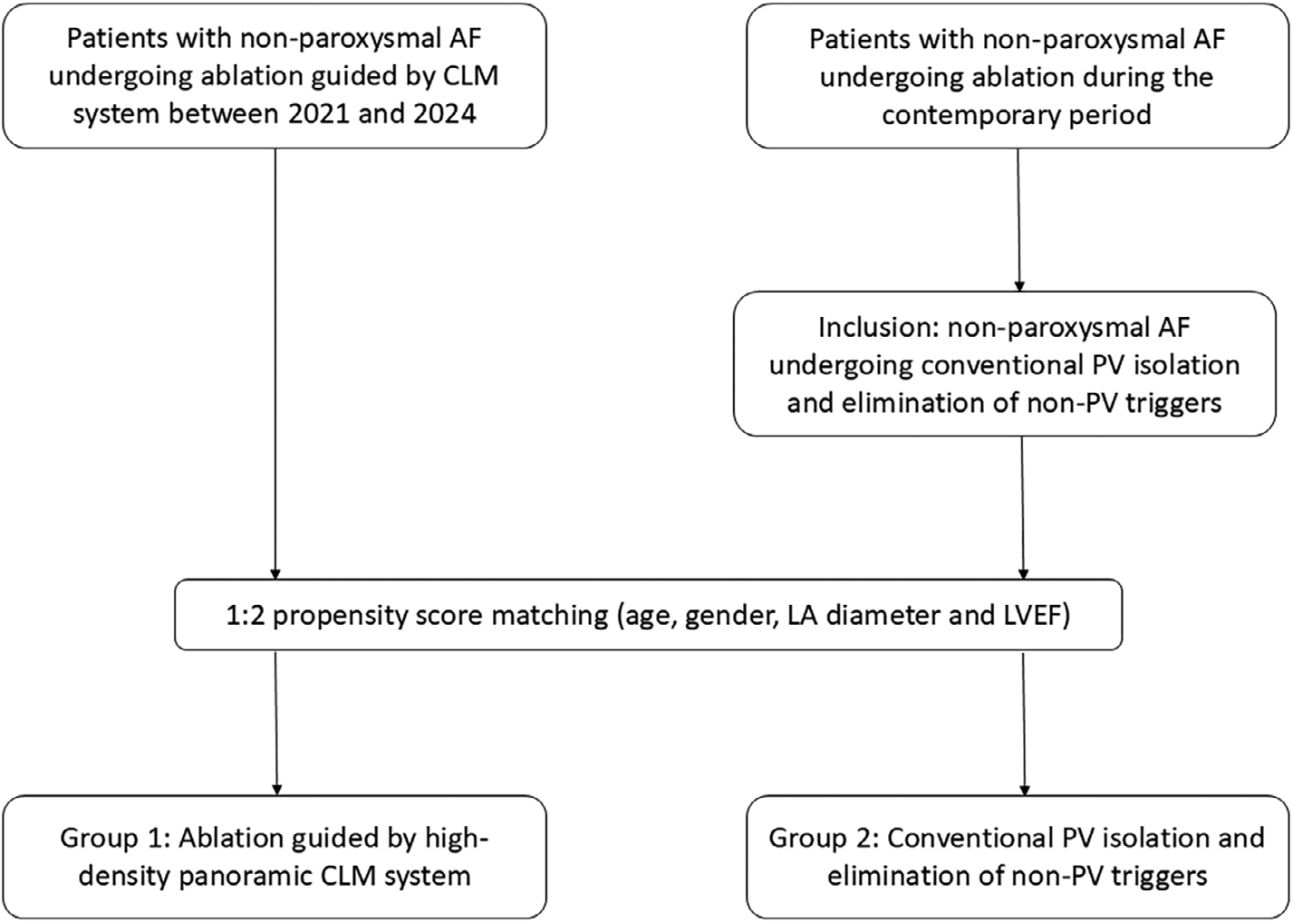
Enrollment of study population. AF, atrial fibrillation; CLM, cycle‐length mapping; LA, left atrial; LVEF, left ventricular ejection fraction; PV, pulmonary vein.

 Between January 2021 and March 2024, a total of 31 patients with symptomatic and drug‐refractory non‐paroxysmal AF referred to Taipei Veterans General Hospital for ablation guided by a new high‐density panoramic CLM system were prospectively enrolled. We then retrospectively screened the patients with non‐paroxysmal AF undergoing conventional PV isolation and elimination of non‐PV triggers during the contemporary period as a conventional group. The conventional group, comprising 62 patients, was formed using a 1:2 matching for age, gender, left atrial diameter (LAD), and left ventricular ejection fraction (LVEF), adhering to a 0.2 caliper width in the propensity‐score technique. The four relevant factors have been shown to be associated with recurrence after AF ablation [14, 15, 16, 17]. The result showed two balanced groups (31 patients in Group 1 received ablation guided by a high‐density panoramic CLM system; 62 patients in Group 2 received conventional PV isolation and elimination of non‐PV triggers). Paroxysmal and non‐paroxysmal AF were defined according to the statement from the 2017 Heart Rhythm Society Expert consensus document. Figure 1 shows the enrollment of the study population.

### 2.2 Study Design

This study was conducted at the Taipei Veterans General Hospital in Taipei after approval by the institutional review board of the Taipei Veterans General Hospital (IRB: 2021‐08‐008B). Written informed consent was obtained from all patients. The long‐term recurrence of AF, atrial flutter, and atrial tachycardia was analyzed.

### 2.3 Electrophysiological Study

An electrophysiological study and catheter ablation in the fasting state were performed in each patient after obtaining informed consent. All antiarrhythmic drugs, except for amiodarone, were discontinued for at least five half‐lives before initiation of the procedure. Amiodarone was held 2 weeks before the procedure. None of the patients received amiodarone during the electrophysiological procedure. Electroanatomical mapping was performed in all patients, and we employed a stepwise procedure of catheter ablation, which has been described in detail before [9].

### 2.4 Identification of Local AF Cycle Length and Spatiotemporal Stability With Cycle‐Length Mapping (CLM) Module

Traditionally, CFAEs were generally thought to be present when any of the following criteria apply: (a) continuous electrical activity, (b) at least two deflections, (c) CL <120 ms, and (d) amplitude >0.05 mV to distinguish genuine fractionation from low‐level artefact [18]. The variation in CFAE CL could be due to the complex interplay of multiple mechanisms contributing to AF. These mechanisms can include reentrant wavefronts, rotors, and wavebreak. In our previous study of persistent AF ablation, the lower temporal variability of the continuous CFAEs was associated with procedural AF termination [11]. In another animal study, regions of highly recurrent electrogram morphology with low CL could be more reflective of arrhythmogenic substrate for AF [19]. Consequently, regions with shorter mean CLs and low variability identified in CLM system were thought to be potential zones of AF drivers.

The CLM system automatically determines the bipolar intracardiac (IC) CL per electroanatomical point and displays it on the reconstruction according to a color scale (mean CL value coloring and standard deviation coloring). At each location, the mapping catheter was maintained in a stable position for a minimum of 10 s to establish the temporal stability of extracted features. When the catheter was not stable for 10 s, acquisitions were repeated. CL of local atrial activity was measured for each point acquired by the mapping catheter and was calculated for every 2.5 s, same as the acquisition time window in the CARTO module for CFAEs and Spatio‐Temporal Dispersion of Electrograms analysis [20, 21]. Voltage peaks greater than the threshold of 0.05 mV were annotated. The intervals between successive peaks were then analyzed. The mean and standard deviation CLs are calculated and displayed as two new coloring types: Cycle‐Length Mean Map and Cycle‐Length Standard Deviation (CLSTD) Map. According to the previous study, regions with local atrial electrogram during AF with mean CL ≤ 140 ms and CLSTD ≤ 30 were thought to be potential drivers of AF [12].

### 2.5 Group 1—Cycle‐Length Mapping (CLM) Method

#### 2.5.1 Step 1: Fast Anatomical Mapping (FAM) and CLM of LA

**FIGURE 2:**
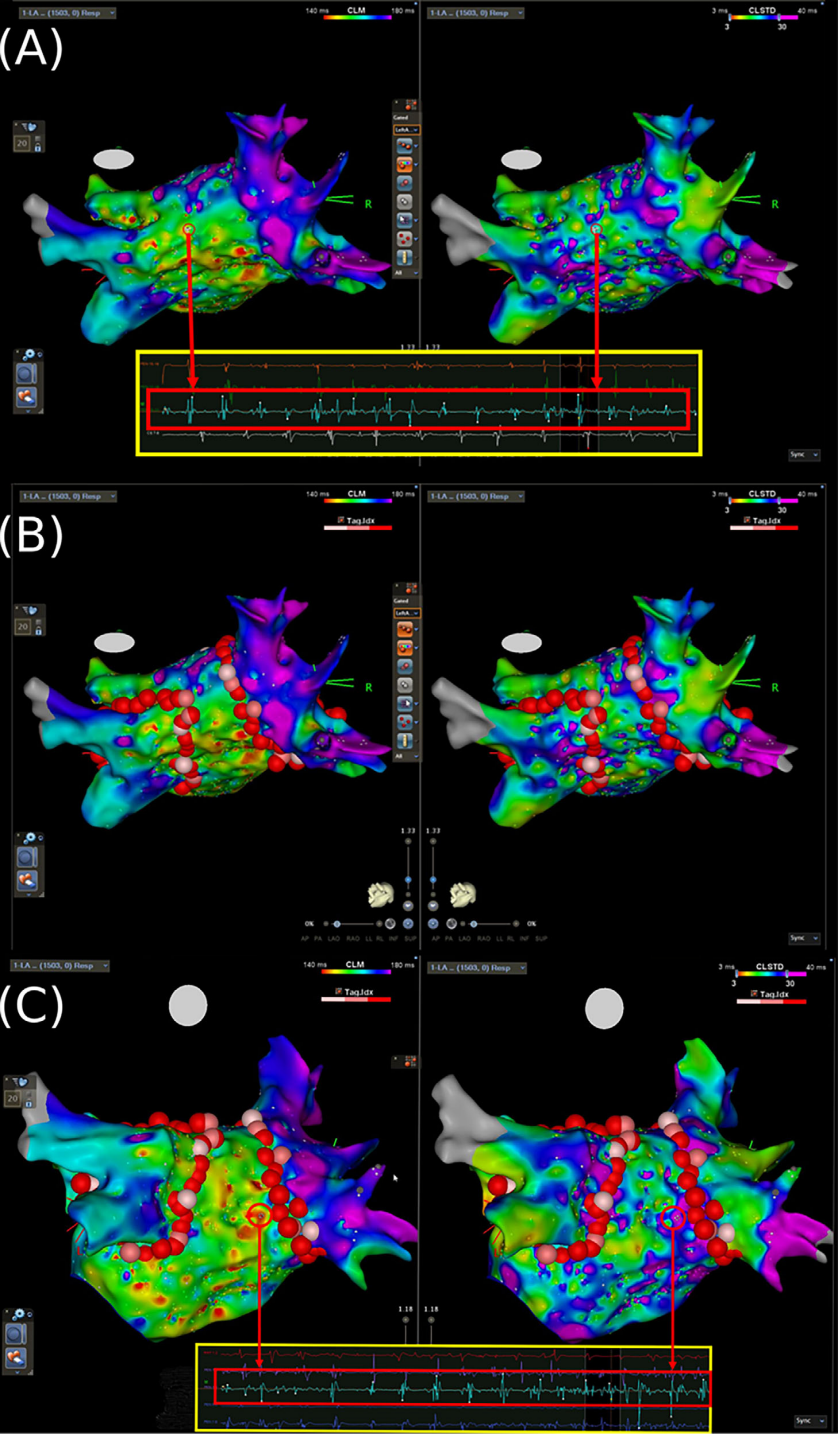
Cycle‐length mapping (CLM)‐guided approach of wide pulmonary antral isolation. (A) After the creation of the FAM, the panoramic mapping system using the CLM module to identify the potential drives was performed. Red arrow indicated the local bipolar atrial signals. (B) The modified ablation line, encircling the short [mean CL ≤ 140 ms] and stable [CLSTD ≤ 30] local AFCL identified by CLM system around the PV antra. (C) Regions with short unstable [CLSTD > 30] local AFCL will not be targeted. Red arrow indicated the local bipolar atrial signals. CLM, cycle‐length mapping; CLSTD, cycle‐length standard deviation; FAM, Fast anatomical mapping. [Colour figure can be viewed at wileyonlinelibrary.com]

After trans‐septal puncture under the guidance of right atriography, a FAM map of the LA and the PVs was created with a multipolar mapping catheter (PentaRay, Biosense Webster). After the creation of the FAM, the panoramic mapping system using the CLM module to identify the potential drives was performed with the PentaRay catheter in the LA (Figure 2A).

#### 2.5.2 Step 2: Modified Wide Antral PV Isolation With Encompassing CLM‐Identified Potential Drivers Around the PV Antra

Wide antral PV Isolation was performed with an irrigated‐tip force‐sensing ablation catheter by ablation at the PV antrum, confirmed by the elimination of the local signal. Radiofrequency energy up to 25–50 W was applied for 10–40 s for each lesion, with a target temperature below 40°C. For NavX system, the target Lesion Index (LSI) was 5.0 for anterior wall and 4.5 for posterior wall [22]. For Carto 3 system, the target Ablation Index (AI) was 500 for anterior wall and 450 for posterior wall [23]. If CLM‐identified potential drivers were located around the PVs, the isolation line would be adjusted to encircle lesions around the PV antra to encompass these CLM‐identified potential drivers (Figure 2B).

#### 2.5.3 Step 3: Potential AF Driver Ablation Using a High‐Density Panoramic Mapping System With CLM Module

If the AF persisted after step 2, CLM mapping was performed again, and elimination of CLM‐identified potential drivers was attempted except for those inside the LA appendage (LAA). Only short and stable AFCL (mean CL ≤ 140 ms and CLSTD ≤ 30) will be targeted. Regions with unstable AFCL (CLSTD > 30) will be ignored (Figure 2C). Ablation at potential driver sites was delivered with a power of 25 to 40 W, with target AI for 400, aiming for the center of the areas. Further ablation was delivered in a cluster of lesions surrounding the first point. Ablation in any region was stopped if AF was terminated or there was no signal remaining in the area of the potential drivers. The ablation time in any 1 cluster was restricted to 5 min. Care was taken not to form a linear lesion, so as not to impact any AF mechanisms in this way. Aside from isolating PVs and targeting potential drivers, no more additional ablation was performed during AF. If the AF organized into an atrial tachycardia or atrial flutter, this was mapped and ablated. If AF even persisted after the above procedures, sinus rhythm was restored with electrical cardioversion.

A right atrial cavotricuspid isthmus (CTI) ablation was routinely performed at the end of the AF procedure. Bidirectional conduction block of linear ablation was demonstrated during sinus rhythm.

### 2.6 Group 2—Conventional Method

#### 2.6.1 Step 1: Wide Antral Pulmonary Vein (PV) Isolation

After trans‐septal puncture under the guidance of right atriography, a decapolar circular or multielectrode catheter was placed in the left atrium (LA) through femoral venous access. The electroanatomic geometry of the LA was constructed using a 3D navigation system (NavX system from Abbott Medical, Minnetonka, MN, USA; or Carto 3 System from Biosense Webster, Diamond Bar, CA, USA). Continuous circumferential lesions were created by encircling the atrial side of the bilateral PV antra with an irrigated‐tip force‐sensing ablation catheter. Radiofrequency energy up to 25–50 W was applied for 10–40 s for each lesion, with a target temperature below 40°C. For NavX system, the target LSI was 5.0 for anterior wall and 4.5 for posterior wall. For Carto 3 system, the target AI was 500 for anterior wall and 450 for posterior wall. If the AF persisted after PV isolation, sinus rhythm was restored using electric cardioversion.

### 2.7 Step 2: Non‐PV Trigger Ablation

After the restoration of sinus rhythm, spontaneous onsets of atrial ectopic beats were recorded, or repeat short runs of sustained AF would be facilitated to initiate AF with or without isoproterenol infusion (4–8 µg/min). Intermittent overdrive pacing from the right atrium (RA), coronary sinus (CS), and PV or administration of intravenous high‐dose adenosine (18–24 mg) were performed to induce atrial ectopy in patients without initial atrial ectopy or ectopy that was non‐inducible with isoproterenol infusion (4–8 µg/min). Continued overdrive pacing was performed until sustained AF was induced.

The location of non‐PV ectopy was evaluated using the activation sequence of the high RA, His‐bundle area, and coronary sinus. Measuring the time difference between the high RA and His‐bundle area activation during sinus rhythm and ectopy (ectopic beat <0 ms) can help reveal the site of ectopy, differentiating between the superior vena cava (SVC), upper crista terminalis, and PVs. For the mapping of non‐PV triggers from the LA, if the earliest activation site was near the left PV ostium or posterolateral portion of the mitral annulus, differential pacing was performed to differentiate the ectopic beats from the vein of Marshall (VOM). Catheter ablation was conducted toward the earliest electrical activity or a local unipolar QS pattern of the ectopic beats preceding the onset of AF. The endpoint of the non‐PV trigger ablation was the disconnection between the SVC and RA, as well as between the coronary sinus and RA, and the elimination of other non‐PV ectopic beats with the negative provocation of AF.

A right atrial CTI ablation was routinely performed at the end of the AF procedure. Bidirectional conduction block of linear ablation was demonstrated during sinus rhythm.

### 2.8 Follow‐Up of AF Recurrence

After discharge, the patients were followed (2 weeks after the catheter ablation, then every 1 to 3 months) at our cardiology clinic or with the referring physician. During each follow‐up, 24‐hour Holter monitoring, or 7‐day event recorder was performed for a week. The primary endpoints were recurrence of all atrial arrhythmia, including AF, atrial flutter, and atrial tachycardia. The recurrent AF, atrial flutter, and atrial tachycardia, and were defined as an episode lasting > 30 s, 3 months after the ablation.

### 2.9 Statistical Analysis

Patient characteristics are expressed as mean ± standard deviation for continuous variables, and as frequency (percentage) for categorical variables. Continuous and categorical variables were compared using the Student's *t* test and the *χ* ^2^ test with Yates’ correction, respectively. Proportions were compared using the *χ* ^2^ test or Fisher's exact test, as appropriate. One‐way analysis of variance was used to compare data among two groups. Propensity‐score matching was used to control for confounders [16, 17]. The propensity score was obtained using logistic regression. The recurrence‐free survival curve was examined using the Kaplan–Meier method with the log‐rank test. Univariable analysis was performed with a Cox regression model, and variables with *p* < 0.05 were taken into further multivariable analysis. Multivariable analysis was performed with a Cox regression model, with results expressed as Hazard ratios (HRs) with 95% confidence intervals (95% CIs). The final statistical significance was set at *p* < 0.05. Statistical significance was set at *p* < 0.05. Statistical analyses were performed using IBM SPSS 26 for Windows (SPSS, Inc., Chicago, IL).

## 3 Results

### 3.1 Baseline Characteristics and Between Two Propensity Score‐Matching Groups With Non‐Paroxysmal AF

The baseline characteristics and demographics of the two groups are shown in Table 1. The age, gender, body mass index (BMI), LAD, LVEF, AF duration, long‐standing persistent AF, and CHA_2_DS_2_‐VASc score were similar between the two groups. In Group 1, a mean of 1.5 ± 1.3 extra‐PV regions (range from 1 to 7) with CLM‐identified potential drivers were observed per patient.

**TABLE 1:** Baseline characteristics and demographics between two groups.

	Group 1 (*n* = 31)	Group 2 (*n* = 62)	*p* value
Age (years)	60.5 ± 11.8	60.6 ± 11.9	0.965
BMI (kg/m^2^)	27.0 ± 4.4	26.1 ± 5.0	0.388
Male (*n*, %)	27 (84.4%)	50 (80.6%)	0.781
AF duration (months)	34.5 ± 30	28.7 ± 23	0.524
Long‐standing persistent AF (*n*, %)	13 (41.9%)	22 (35.5%)	0.651
Diabetes mellitus (*n*, %)	4 (12.9%)	9 (14.5%)	1.000
CHF (*n*, %)	8 (25.8%)	6 (9.7%)	0.063
Hypertension (*n*, %)	12 (38.7%)	32 (51.6%)	0.276
CAD (*n*, %)	10 (32.3%)	11 (17.7%)	0.124
Old stroke/TIA (*n*, %)	2 (6.5%)	9 (14.5%)	0.326
Hyperthyroidism (*n*, %)	2 (6.5%)	5 (8.1%)	1.000
Hyperlipidemia (*n*, %)	5 (16.1%)	17 (27.4%)	0.303
Chronic Kidney Disease (*n*, %)	0 (0%)	5 (8.1%)	0.165
CHA_2_DS_2_‐VASc score	1.81 ± 1.7	1.95 ± 1.8	0.707
Left atrial diameter (mm)	42.8 ± 5.3	42.9 ± 5.7	0.956
Left ventricular ejection fraction (%)	53.4 ± 11.2	53.4 ± 8.1	0.999

Abbreviations: AAD, anti‐arrhythmic drug; AT, atrial tachycardia; BMI, body mass index; CAD, coronary artery disease; CHF, congestive heart failure; TIA, transient ischemic attack.

**FIGURE 3:**
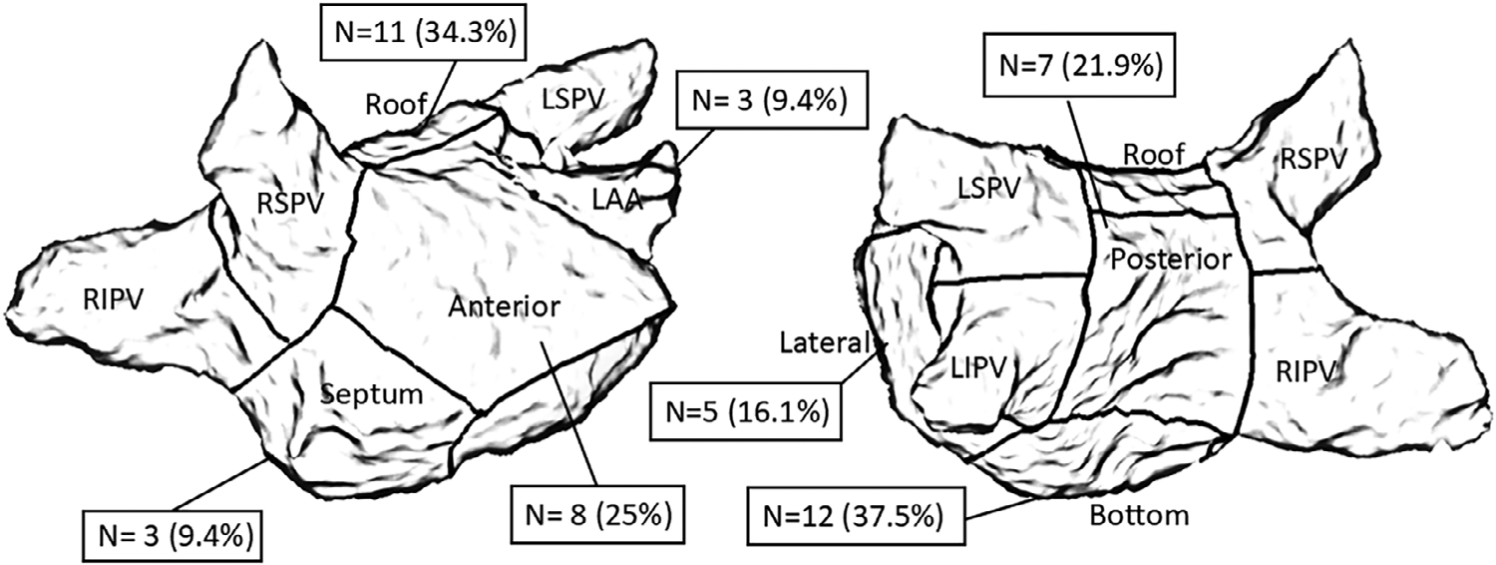
The distribution of CLM‐identified potential AF drivers in different regions of LA chamber in Group 1. (N: Number of patients). AF, atrial fibrillation; CLM, cycle‐length mapping; LA, left atrial; LAA, left atrial appendage.

The distribution of CLM‐identified potential drivers in different regions of LA chamber in Group 1 was demonstrated in Figure 3. The percentage of patients in Group 1 with potential drivers detected in each region was calculated. The most common area detected with potential drivers was the roof (11 patients, 34%), followed by the LA anterior wall (8 patients, 25%), and the LA posterior wall (7 patients, 22%). In Group 2, a mean of 0.3 ± 0.5 non‐PV triggers were identified after the restoration of sinus rhythm with PV isolation or cardioversion. Twelve (19.4%) patients had non‐PV triggers, including 1 (1.6%) in SVC, 1 (1.6%) in coronary sinus (CS) ostium, 5 (8.2%) in VOM, 1 (1.6%) in LA septum, 4 (6.6%) in the posterior wall and 6 (9.8%) in the anterior wall or LA appendage base.

In the whole study population, patients with long‐standing persistent AF had more non‐PV ablation sites (a mean of 1.0 ± 1.2) than those with persistent AF (a mean of 0.6 ± 0.8, *p* = 0.038 for long‐standing persistent AF vs. persistent AF).

### 3.2 Procedural Details and Response to Radiofrequency Catheter Ablation

During catheter ablation of AF, PV isolation was achieved in all patients of both groups. In Group 1, the shortest and stable AFCL is located in LA septum before PV isolation. After PV isolation, repeated CLM of LA chamber in Group 1 showed the fastest and stable AFCL over the roof, and there were no significant changes of AFCL over all LA chambers after PV isolation (Table 2). During ablation, procedural termination of AF could be observed in 7 (22.6%) patients of Group 1 and 6 (9.7%) patients of Group 2 (*p* = 0.137) (Table 3).

**TABLE 2:** Shortest and stable AF cycle length within LA chamber before and after PVI.

	Before PVI	After PVI	*p* value
Right superior pulmonary vein (ms)	152 ± 21	−	−
Right inferior pulmonary vein (ms)	147 ± 20	−	−
Left superior pulmonary vein (ms)	148 ± 17	−	−
Left inferior pulmonary vein (ms)	148 ±19	−	−
Left atrial roof (ms)	146 ± 18	128 ± 58	0.136
Left atrial posterior wall (ms)	142 ± 16	135 ± 53	0.555
Left atrial anterior wall (ms)	141 ± 14	144 ± 19	0.438
Left atrial septum (ms)	139 ± 14	147 ± 20	0.095
Left atrial bottom (ms)	148 ± 21	147 ± 18	0.807
Left atrial appendage base (ms)	149 ± 19	146 ± 34	0.705

*Note*: Stable AF cycle length: CLSTD (cycle‐length standard deviation) <30.

Abbreviations: AF, atrial fibrillation; LA, left atrial; PVI, pulmonary vein isolation.

**TABLE 3:** Procedural details and long‐term outcome between two groups.

	Group 1 (*n* = 31)	Group 2 (*n* = 62)	*p* value
Procedural termination of atrial fibrillation (*n*, %)	7 (22.6%)	6 (9.7%)	0.137
Complications	1 (3.2%)	1 (1.6%)	0.618
Tamponade (*n*, %)	0 (0%)	0 (0%)	−
Hematoma/Pseudoaneurysm (*n*, %)	1 (3.2%)	1 (1.6%)	−
Stroke/TIA (*n*, %)	0 (0%)	0 (0%)	−
Procedural time (min)	229 ± 63	177 ± 54	0.004
Fluoroscopy time (min)	44.8 ± 13.3	35.7 ± 17	0.074
Recurrence of all atrial arrhythmia (*n*, %)	10 (32.3%)	39 (62.9%)	0.037
Recurrence of atrial fibrillation (*n*, %)	9 (29.0%)	33 (53.2%)	0.101
Recurrence of atrial Flutter or AT (*n*, %)	4 (12.9%)	11 (17.7%)	0.709
Anti‐arrhythmic drugs during follow‐up			
Class I AAD	4 (12.9%)	14 (22.6%)	0.404
Class II AAD	10 (32.3%)	25 (40.3%)	0.502
Class III AAD	6 (19.4%)	22 (35.5%)	0.151
Class IV AAD	3 (9.7%)	6 (9.7%)	1.000

Abbreviations: AAD, anti‐arrhythmic drug; AT, atrial tachycardia; PV, pulmonary vein; TIA, transient ischemic attack.

**FIGURE 4:**
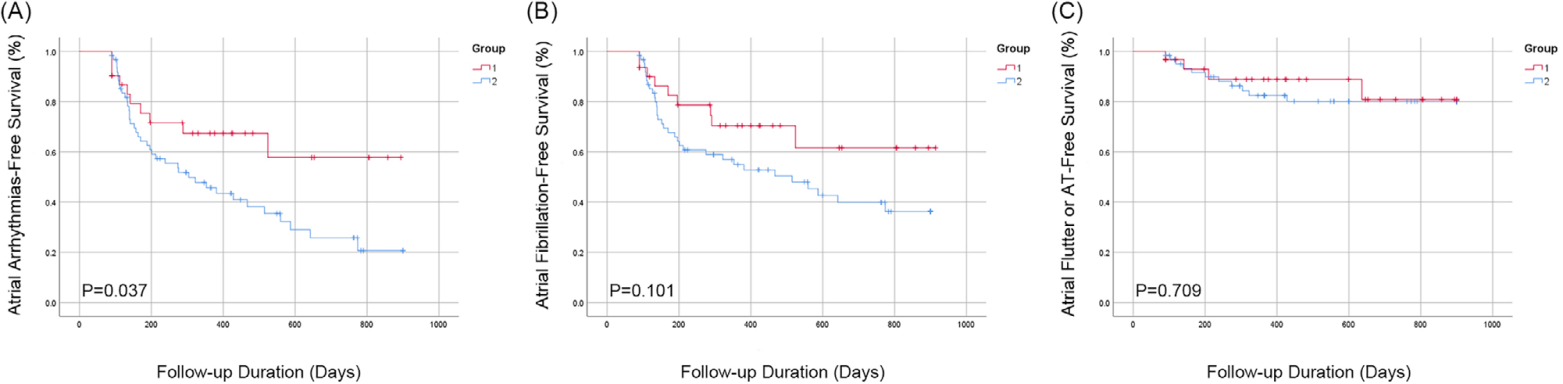
Kaplan–Meier curve of recurrence after the index procedure during follow‐up between the two groups. (A) Recurrence of atrial arrhythmias between Group 1 (CLM method) and Group 2 (conventional method), log‐rank *p* = 0.37. (B) Recurrence of atrial fibrillation between Group 1 (CLM method) and Group 2 (conventional method), log‐rank *p* = 0.101. (C) Recurrence of atrial flutter or atrial tachycardia between Group 1 (CLM method) and Group 2 (conventional method), log‐rank *p* = 0.709. CLM, cycle‐length mapping. [Colour figure can be viewed at wileyonlinelibrary.com]

1. **Safety characteristics between two groups**
  The overall major complication rate was low and comparable between the two groups, with 1 (3.2%) hematoma/pseudoaneurysm in Group 1 and 1 (1.6%) hematoma/pseudoaneurysm in Group 2. The safety characteristics of the two groups are shown in Table 3.
2. **Long‐term outcome during clinical follow‐up**
  With a mean follow‐up of 23.2 ± 5.1 months, patients in Group 1 had a lower recurrent atrial arrhythmia than those in Group 2 did (10 [32.3%] patients in Group 1 and 39 [62.9%] patients in Group 2, Log Rank *p* = 0.037, Figure 4A), that was driven by reduced recurrent AF (9 [29%] patients in Group 1 and 33 [53.2%] patients in Group 2, Log Rank *p* = 0.101, Figure 4B). The anti‐arrhythmic drugs between the two groups were similar (Table 1). There was no difference in recurrence of atrial flutter or atrial tachycardia (4 [12.9%] patients in Group 1 and 11 [17.7%] patients in Group 2, Log Rank *p* = 0.709, Figure 4C).
3. **Predictors of long‐term outcome**
  In the univariate Cox regression analysis, age, AF duration, Type 2 DM, LAD, and ablation method could predict long‐term recurrent atrial arrhythmias. In the multivariate Cox regression analysis, PV isolation, along with AF driver ablation using CLM system was the only independent predictors of recurrent atrial arrhythmia after catheter ablation of non‐paroxysmal AF (Table 4).

**TABLE 4:** Univariate and multivariate analyses of recurrent atrial arrhythmias after catheter ablation.

	Univariate analysis		Multivariate analysis	
	*p* value	95% CI	*p* value	95% CI
Groups	**0.042**	**0.242–0.974**	**0.011**	**0.150**–**0.782**
Age	**0.039**	**1.001–1.054**	0.412	0.983–1.043
AF duration	**0.016**	**1.001**–**1.012**	0.059	1.000–1.043
CHA2DS2–VASc score	0.174	0.953–1.309		
Male	0.770	0.449–1.811		
BMI	0.644	0.962–1.064		
Diabetes mellitus	**0.013**	**1.221**–**5.385**	0.234	0.693–4.472
Congestive heart failure	0.734	0.390–1.939		
Old stroke/TIA	0.202	0.756–3.758		
Hypertension	0.827	0.607–1.867		
Coronary artery disease	0.749	0.412–1.891		
Chronic kidney disease	0.083	0.888–0.6991		
Hyperthyroidism	0.555	0.219–2.265		
Hyperlipidemia	0.284	0.326–1.388		
Left ventricular ejection fraction	0.228	0.957–1.010		
Left atrial diameter	**0.037**	**1.004**–**1.128**	0.098	0.991–1.119

Abbreviations: AF, atrial fibrillation; BMI, body mass index; CAD, coronary artery disease; CHF, congestive heart failure; TIA, transient ischemic attack.

The *p* value was considered significant when *p* < 0.05.

## 4 Discussion

The main findings of this study were as followed: (1) High‐density panoramic CLM mapping could identify potential AF drivers and assist in modifying the wide‐area PV ablation by encompassing CLM‐identified drivers around the PV antra. (2) As compared with the conventional PV isolation and elimination of non‐PV triggers, the stepwise approach of the CLM method resulted in less recurrent atrial arrhythmias in patients with non‐paroxysmal AF. (3) PV isolation, along with potential AF driver ablation using the panoramic CLM system, was the independent predictor of recurrent atrial arrhythmias after catheter ablation of non‐paroxysmal AF.

### 4.1 The Potential Therapeutic Target of Patients With Non‐Paroxysmal AF Beyond the PV Isolation

Previous investigations have suggested that regions exhibiting short dominant AFCL might serve as individualized targets for catheter ablation in patients with persistent AF [24, 25]. Shiomyo et al. further demonstrated that the rate of AF termination improved when ablation was extended to sites identified as having dominant AFCLs using manually curated local activation time maps derived from the CARTOFINDER system [26]. While the precise electrophysiological mechanisms underlying AF drivers remain a topic of ongoing debate, they are commonly hypothesized to include reentrant circuits, rotors, focal impulses, or a heterogeneous interplay among these elements. Notably, Hansen et al. proposed that human AF may be sustained by intramural microanatomic reentry circuits, which manifest on the endocardial surface as stable or unstable rotors, focal discharges, or breakthrough activations—phenomena driven by the complex three‐dimensional architecture of the atrial wall [27]. In our study, we found that PV isolation combined with substrate modification targeting areas of short and relatively stable AFCL was associated with improved long‐term clinical outcomes.

### 4.2 Balanced Substrate Modification by Targeting Potential AF Drivers Detected in CLM System

As for WACA performed in the first step, the PV antrum was the area extending 5 mm from the PV ostium, but consistent identification could be an issue [28]. In some cases, the isolation may be too small in the anterior PV segments, and conversely, the ablation area might be too extensive in the posterior and roof regions, potentially causing connections between the isolated areas [28]. With CLM guidance, if these CLM‐identified potential drivers were in the PV vicinity, the isolation line of WACA could be modified to encompass the potential drivers, in order to have more efficacy with WACA.

Although WACA targeting the PVs may be insufficient as a standalone strategy in patients with non‐paroxysmal AF, the addition of extensive extra‐PV substrate modification has been associated with increased atrial scarring and a heightened risk of procedural complications. Gibson et al. reported that pulmonary hypertension occurred in 1.4% of patients post‐ablation despite the absence of PV stenosis, and this was correlated with elevated left atrial pressure, larger atrial scar burden, diabetes mellitus, and obstructive sleep apnea [29]. Consistent with this, the DECAAF (Delayed Enhancement—MRI Determinant of Successful Catheter Ablation of Atrial Fibrillation) study demonstrated that greater degrees of atrial fibrosis were predictive of AF recurrence following ablation [30]. These findings underscore the importance of achieving an optimal balance in substrate modification—adequate enough to reduce arrhythmogenic potential, yet conservative enough to minimize structural damage and procedural risk, thereby improving long‐term clinical outcomes.

With the innovative CLM algorithm, our results showed that efficient identification of potential drivers of AF could be achieved and be ablated effectively. Limited and focused substrate modification guided by the CLM system might decrease tissue damage and result in less subsequent LA scar formation and possible procedure‐related complications.

### 4.3 Future Perspectives of Ablation of Non‐Paroxysmal AF

Clinical outcomes of non‐paroxysmal AF ablation using linear lesions or substrate modification through electrogram defragmentation have generally been modest. Moreover, these approaches may necessitate extensive ablation, potentially leading to adverse consequences such as impaired atrial hemodynamics and proarrhythmic effects [31]. These limitations underscore the pressing need for innovative ablation strategies aimed at targeting individualized AF drivers.

To our knowledge, this study represents the first to evaluate the long‐term outcomes of non‐paroxysmal AF ablation guided by the new CLM system using a propensity score‐matched cohort. Real‐time, high‐density endocardial contact mapping of electrograms during ongoing AF enables the identification of additional putative AF drivers. Furthermore, the panoramic high‐density CLM system facilitates refinement of wide‐area circumferential PV isolation by delineating and encompassing potential driver regions within the PV antra. Compared to conventional wide‐area circumferential PV isolation combined with empirical non‐PV trigger ablation, the stepwise CLM‐guided approach was associated with a significantly lower rate of recurrent atrial arrhythmias in patients with non‐paroxysmal AF. These findings support the rationale for conducting a randomized controlled trial to further investigate the clinical benefits of CLM‐guided AF driver ablation.

### 4.4 Limitation

There are several limitations in this study. (1) The integrated CLM module is a novel software used for catheter ablation in non‐paroxysmal AF. The clinical efficacy and safety were still under investigation. We have discussed efficacy and safety in the present study with patients in the clinics. Patients who agreed to join this study and were willing to have at least 12‐month follow‐up were enrolled. Therefore, this study was a non‐consecutive enrollment. (2) Although we used the reverse U curve to locate the Penta‐Ray into the septum and the bottom, which improved the stability in these areas, it was still possible to overestimate/underestimate the CLM‐identified potential AF drivers. (3) Because of prolonged procedure time with CLM map, RA mapping was not routinely performed. The arrhythmogenicity in the RA might be underestimated. (4) Some patients were followed with a 24‐h Holter, and others were followed with 1‐week recordings of a cardiac event recorder. The inconsistent ECG monitoring during follow‐up might impact the recurrence. In this study, we followed patients regularly in the clinics and evaluated the symptoms of patients.

## 5 Conclusion

Identification of the potential extra PV drivers in non‐paroxysmal AF is crucial. The CLM system can assist in modifying the wide‐area isolation around the PV antra. Compared to the conventional method, the application of CLM might have better outcomes in patients with non‐paroxysmal AF.

## Conflicts of Interest

The authors declare no conflicts of interest.

## Data Availability

The original contributions presented in the study are included in the article/Supplementary Material, further inquiries can be directed to the corresponding author.
